# Intratendinous Patellar Ganglion Cyst with Coexistant Osgood Schlatter Disease

**DOI:** 10.5334/jbr-btr.1195

**Published:** 2016-10-27

**Authors:** Wouter Mebis, Tjeerd Jager, Eddy Van Hedent

**Affiliations:** 1 Algemeen Stedelijk Ziekenhuis Aalst, BE

**Keywords:** Osgood Schlatter Disease, intratendinous cyst, ganglion, patellar tendon, ultrasound, magnetic resonance

## Case

**Figure 1:**
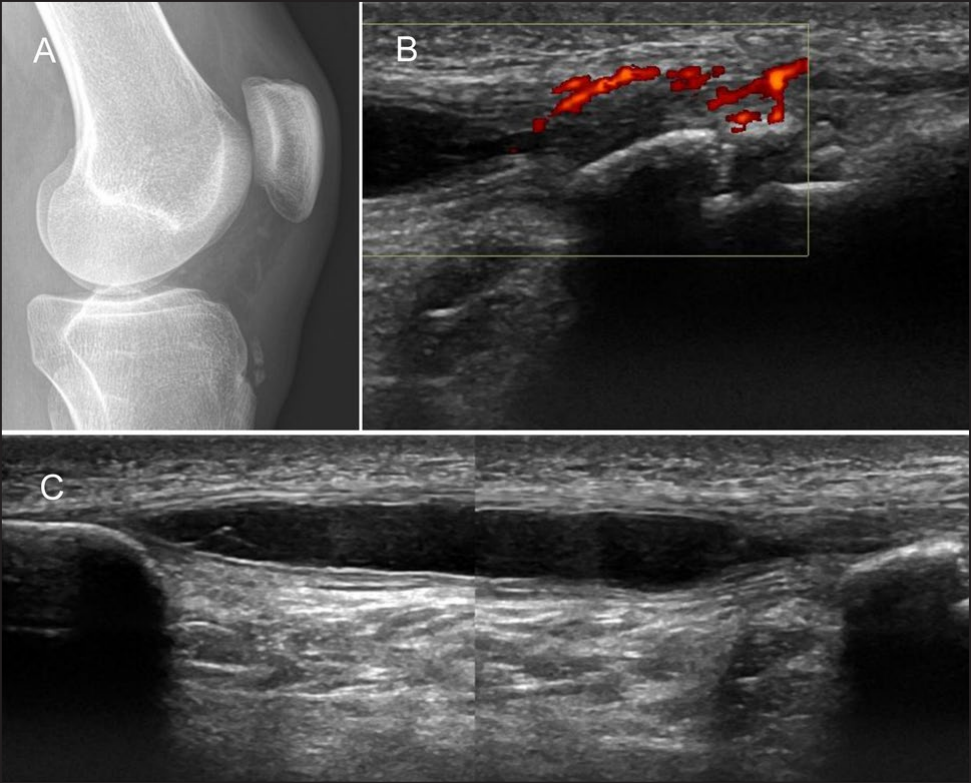
**(A)** Lateral X-ray of the knee; **(B & C)** High frequency ultrasound of the patellar tendon, sagittal plane.

A 19-year-old boy was sent in by the general practitioner with complaints of persisting tenderness and swelling just below the knee. Conventional radiography of the knee showed fragmentation of the tibial tuberositas pointing towards Osgood Schlatter Disease (Figure 1A).

Ultrasound examination of the knee with a high frequency linear probe was performed. Findings included fragmentation of the tibial tuberosity, hypoechoic tendon and increased power doppler signal, compatible with Osgood Schlatter disease (Figure 1B). Additionally, a sharply delineated, elongated anechoic structure with enhanced through transmission occupied the center of the patellar tendon (Figure 1C). A small pedicle towards the proximal bony fragment of the tibial tuberosity seemed to be present. The diagnosis of an intratendinous patellar ganglion cyst was made, possibly related to a coexistant chronic Osgood-Schlatter disease.

**Figure 2:**
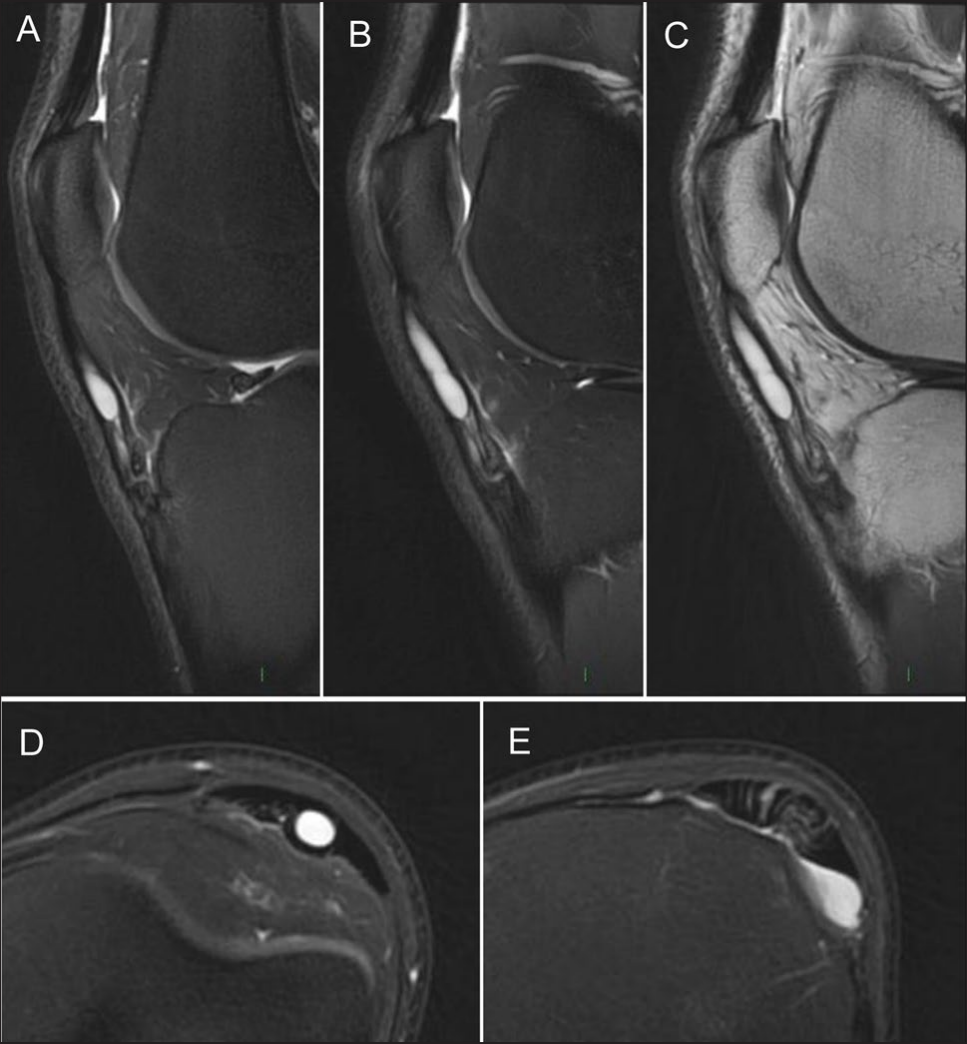
**(A & B)** 3T MR of the knee with sagittal T2-WI **(C)** PD-WI and **(D)** axial T2-WI at the level of the cyst **(E)** the distal tendon insertion.

To further investigate the etiology of the intratendinous ganglion cyst additional magnetic resonance imaging (MR) was performed (Figure 2). This demonstrated swelling and increased signal intensity of the distal patellar tendon on both T2-weighted images (WI) and intermediate/proton density weighted images (PD-WI) with fragmentation of the tibial tuberosity as part of Osgood Schlatter disease. A large intratendinous cyst with high signal intensity on T2- and PD-WI occupied nearly the whole length in the centre of the tendon and terminated into a thin pedicle towards the proximal bony fragment. Additionally, there was mild fluid distension of the deep infrapatellar bursa. The diagnosis of an intratendinous patellar ganglion cyst with a coexistant chronic Osgood-Schlatter disease was confirmed.

## Comment

Osgood-Schlatter disease (OSD) is caused by repetitive microtrauma to the apophysis of the tibial tuberosity which leads to apophysitis and distal patellar insertional tendinopathy. OSD typically affects active children aged 11–15, especially those practicing sports with a lot of jumping and/or kicking. Boys are more often affected than girls and generally there is no history of a specific injury as most cases are caused by repeated overuse. Clinical examination shows pain, tenderness and swelling at the tibial tuberosity. Activities that involve jumping or kicking aggravate the pain [1,2].

A conventional lateral radiograph of the knee may show soft tissue swelling at the tibial tuberosity and/or fragmentation of the ossification centre [1,2].

Ultrasound examination also demonstrates fragmentation of the apophysis and hypoechoic swelling of the physeal cartilage. Moreover, small calcifications and bony irregularities related to osteochondrosis may be seen. Power or colour Doppler may reveal local hyperemia. In more advanced chronic disease, thickening and hypoechoic degenerative changes of the distal patellar tendon can be present. Deep infrapatellar bursitis may occur in severe cases [1,2].

MR imaging can clearly reveal patellar tendonitis with thickening and heterogeneous signal reflecting tendon oedema. There may be soft tissue swelling and focal bone marrow edema at the tibial tuberosity. Deep infrapatellar bursitis can also be demonstrated easily [3].

Intratendinous cyst formation can be easily depicted on both ultrasound and MR but has only been scarcely reported in literature, especially those involving the patellar tendon. However, it is generally accepted that they arise from mucoid degeneration of intratendinous collagen fibers due to repetitive trauma, which also causes OSD [3]. We therefore think it is safe to assume that there is a relation between both entities.
